# Is there a beneft in maintaining printed and online versions of scientific journals? *ABO* is going 100% online

**DOI:** 10.5935/0004-2749.2023-1001

**Published:** 2023

**Authors:** Newton Kara-Junior

**Affiliations:** 1 Faculdade de Medicina, Universidade de São Paulo, São Paulo, SP, Brazil

Before the Internet made it possible to transport large amounts of information and accelerate the transfer of knowledge, online journals were undervalued and considered inferior to print editions. With technological evolution, the scenario has changed, and currently, online journals have immense potential because of the advantages of information technology associated with telecommunications. Technology has enabled researchers and readers to have quick and easy access to published knowledge anywhere worldwide. The Internet has also facilitated the process for submitting, evaluating, and publishing scientific papers. Thus, distances and borders are no longer barriers, and scientific periodicals have become global journals.

Thanks to the exchange of knowledge, powered by online journals, the quality of scientific research has progressed rapidly. Scientific investigations aim to find answers to “doubts” in the literature, thus increasing existing knowledge. In general, high-level research responds to very specific “doubts”, and their interpretation is only possible when the context in which the “question” is inserted is known. Thus, scientific articles from cutting-edge investigations carry fragmented information, which add little to the knowledge of readers who do not master the subject.

Researchers and academics do not consult “journals”, but articles in the “cloud”, through keywords. They select several publications on the topic of interest, critically analyze the scientific methodologies used in the research, establish the value of each article, and, finally, connect the information to add new elements to their knowledge^(1)^.

Despite the success of online editions, some journals maintain two publication formats, i.e., print and digital. The dual channel of diffusion has its value in making scientific knowledge more accessible. However, the more qualified the journal, the more important it is to use information transmission tools only available online. As journals’ index bases do not allow the printed version to be very different from the electronic one, the paper edition can limit the journal’s progress.

The online edition transmits more information and data than the printed version. As it is a digital base, it allows access to parallel information, through links that make it possible to access other channels, such as sites, images, video, and sound. The reader can consult the research database, review previous journal issues, access links that can supplement the subject publication, give instant feedback, and share news. The number of views for each article can be also measured. In the printed edition, there are restrictions and space limitations for text creation. In the electronic publication, there are no page or image limits. In the online version, the frequency or number of articles per issue may be increased, even allowing the publication of articles continuously, without a substantial increase in the publication cost^(2)^.

Another current debate is the controversy regarding the private channel for the diffusion of scientific research because many of the best-known scientific journals, in addition to being exclusively online, have their disclosure rights belonging to big publishers, who profit by charging authors and readers. By contrast, there are journals belonging to institutions and specialist societies that sponsor the entire editorial process, allowing fiscal equilibrium without taxing authors and readers.

The *Brazilian Archives of Ophthalmology (Arquivos Brasileiros de Oftalmologia* - *ABO*), which belongs to the Brazilian Council of Ophthalmology (CBO), is a scientific journal with an excellent impact factor (IF). It does not charge authors to publish or readers to access its contents. Furthermore, by publishing its printed version concomitantly with the online version, it points out that new technologies do not need to replace previous ones if they fulfill different purposes. Thus, when the journal arrives at ophthalmologists’ homes, it helps update a population of readers with no experience in scientific research, who, in general, would not access the Internet version.

We agree with the supporters who maintain the two modes of diffusion to make scientific knowledge more accessible. However, the higher the scientific standard of the journal, measured by its IF, the greater the publication of articles from high-level research, with great citation potential. Advanced research is very specific and is aimed at answering particular questions in the literature and originating articles that add knowledge to readers familiar with that context, who, in most cases, are also researchers. Physicians without research training generally acquire knowledge through scientific literature by reading review articles and meta-analyses, in which authors select and critically analyze sets of articles on the same topic, interpret the results, and draw conclusions that will guide clinical practice.

Owing to the diligence of its current and past editors and reviewers, The *ABO* has reached a status of excellence, publishing articles of great clinical interest and methodological accuracy worldwide. The *ABO* has become a global journal with great diffusion of high-level science. Thus, maintaining the printed publication, despite its history and prestige, currently makes little sense because the information available in the single edition is very specific, fragmented, and static that, alone, it adds little to knowledge acquisition. In *2023*, ABO will be 100% digital.

On the contrary, efforts and investments could be directed toward making online publication more accessible through strategies such as the following: (1) broadcasting, in scientific social networks, of editorials, case reports, review articles, and isolated articles with comments from the editors, which help contextualize the information transmitted; (2) training doctors to answer clinical questions by searching for articles in the digital scientific databases; and (3) training readers for critical analysis of scientific articles.

Considering that those responsible for printed journals have to live with problems of high-printing and postage costs and that Brazilian Ophthalmology is facing important challenges, with the CBO assuming more responsibilities for teaching, medical practice, professional defense of the ophthalmologist, etc., makes it difficult to justify subsidy to a printed version of the ABO. This happens when the benefit to regular readers becomes limited and when there is already a commitment to open access to its content and freedom from publication fee, in addition to contributing to the preservation of the environment, by avoiding printing on paper made from cellulose pulp.

In view on how much the Internet has changed information access and the popularization of smartphones, tablets and notebooks, we can discuss and reevaluate *ABO*’s communication strategy. Given the rapid circulation of scientific knowledge, we believe that we can improve our journal’s efficiency and contribute to a better practice of our readers. Speed and information quality are the gains of ABO being 100% digital in 2023.
